# Plasmon-Induced Graphene/Silicon Schottky Junctions for Ultrasensitive Gas Sensing

**DOI:** 10.1021/acssensors.5c01920

**Published:** 2025-09-30

**Authors:** Katarzyna Drozdowska, Janusz Smulko, Tesfalem Welearegay, Lars Österlund, Sergey Rumyantsev

**Affiliations:** † Department of Metrology and Electronic Systems, Faculty of Electronics, Telecommunications, and Informatics, 49557 Gdańsk University of Technology, G. Narutowicza 11/12, 80-233 Gdańsk, Poland; ‡ Department of Materials Science and Engineering, The Ångström Laboratory, 8097 Uppsala University, P.O. Box 35, SE-75103 Uppsala, Sweden; § 243577 Institute of High Pressure Physics PAS, 01-142 Warsaw, Poland

**Keywords:** gas sensors, graphene, palladium nanoparticles, Schottky junction, localized surface plasmon resonance

## Abstract

Modulating metal oxide-based gas sensors with light is emerging as an alternative to enhance their sensitivity and selectivity. Plasmonic gas sensors based on excitation of the localized surface plasmon resonance (LSPR) in noble metals have recently shown promising properties. The classic approach of incorporating LSPR in sensors is to measure changes in the optical properties of the plasmonic material that depend on the surrounding local environment, i.e., an optical sensor device. The less common approach is to utilize chemiresistive sensors that consist of a gas-sensitive material (like graphene) decorated with plasmonic nanoparticles and employ solely electrical measurements for sensing data acquisition. This work demonstrates a chemiresistive gas sensor graphene/silicon Schottky junction decorated with palladium nanoparticles (PdNPs) that exhibit LSPR in the UV light range. We demonstrate a method of recording responses of plasmonic gas sensors based only on their DC characteristic measurements, which is simplified compared to optical and spectroscopic methods. Supported by the DC characteristics recorded under different UV wavelengths (255 nm, 275 nm, 355 nm), we show that the highest sensitivity to NO_2_ and NH_3_ is obtained for plasmonic resonant wavelength excitation of the PdNPs occurring at about 275 nm. The LSPR-modulated sensor response is nearly 14 times greater for NO_2_ gas compared to NH_3_ and exhibits a sensitivity toward NO_2_ gas with an ultralow detection limit of 4 ppb, thus showing that both the selectivity and sensitivity of plasmonic chemiresistive gas sensors can be significantly enhanced by LSPR-tuned light modulation.

## Graphical abstract

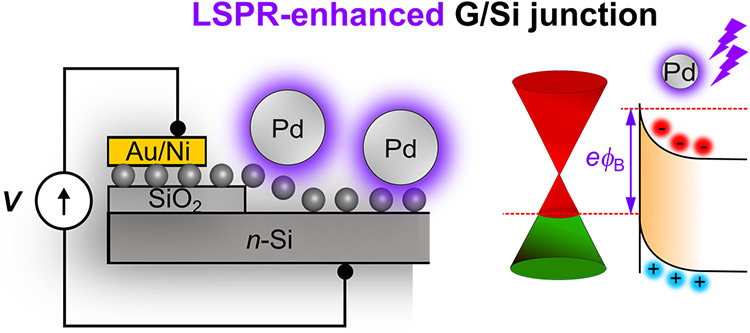

The intrinsic properties of graphene and other two-dimensional materials (2D) make them excellent transducing nanomaterials for gas-sensing applications due to their tunable physicochemical properties.^1,2^ A large fraction of their atoms is exposed at their surfaces, making them sensitive to changes in their environment. When 2D materials are exposed to gas molecules, the adsorption of a single molecule can significantly alter their physical properties.^3^ Owing to its transport properties and high carrier mobility, graphene is an excellent 2D material for chemiresistive gas sensor applications and is particularly suited for low-power consumption devices.^4^ Thus, graphene-based devices have demonstrated the potential for cheap, ultrasensitive, low-power, flexible, and miniaturized sensor devices. Hence, employing graphene as the active part of the sensor in field-effect transistors or Schottky diodes provides additional possibility of modulating the sensing properties with voltage bias, prospectively leading to high sensor performance with lower detection limit and enhanced selectivity.^5,6^ Additionally, the substrate selection for the deposition of graphene can affect the stability of the electrical response to gases (reduced drift in time).^7^ For instance, the Schottky junction between graphene and n-doped silicon prevents graphene from fast aging by adsorbed water molecules.^8,9^ It ensures the repeatability of electrical characteristics compared to field-effect transistors with graphene channels deposited on silicon and its oxide layer (SiO_2_/Si). The graphene structure typically provides a large surface area rich in defects.^10^ Notably, the high density of defects and imperfections of graphene structures improves the adsorption of gas molecules, exhibiting remarkable sensor response, low detection limit, and high selectivity.^11^ The higher sensitivity of defective or doped graphene was also confirmed by theoretical computations.^12,13^

Graphene is intrinsically inert and nonselective, and its gas-sensing property can be enhanced by doping or composing with other nanomaterials of different chemical, electrical, and optical properties. One feasible way of doing it is to effectively control its defectiveness during the synthesis and growth steps and functionalize the surface with noble metal nanoparticles to enhance their sensitivity and selectivity toward target gases.^14,15^ Properties of graphene decorated with noble metal nanoparticles can be modulated with light irradiation.^16^ For instance, graphene/silicon Schottky diodes decorated with gold nanoparticles (AuNPs) of 8 nm diameter exhibited increased sensitivity to chloroform and tetrahydrofuran under yellow light (592 nm), coinciding with the localized plasmon resonance (LSPR) in AuNPs.^17^ LSPR is associated with the collective, geometrically confined displacement of charge within the nanoparticle and depends on the permittivity of the particle morphology and the surrounding medium relative to the nanoparticle.^18^ Thus, plasmonic gas sensors are very promising, particularly when LSPR is induced under ultraviolet (UV) or visible light irradiation. Noble metals such as Au, Ag, Pt, or Pd are the conventional plasmonic materials.^16,19^ These metals also have significant optical losses in the UV–visible region due to d → s interband transitions, which is also an advantage in gas-sensing applications when such metal nanoparticles are hybridized with 2D materials. Thus, light irradiation has been shown to modify adsorption/desorption processes and enhance gas detection properties for graphene-based chemiresistors.^20^ Irradiation with light constitutes an alternative to thermal activation and is particularly attractive for low-power consumption devices when the LEDs of optical powers in the mW, or even μW, range are employed. Both LSPR-induced electric field enhancement and interband absorption induced by UV–visible light are expected to influence the gas-sensing properties of graphene/silicon Schottky junctions. Even though plasmonic nanomaterials offer high sensitivity to gases, the challenge is to simplify the measurement procedure for collecting gas-sensing responses for plasmonic sensors, as they usually rely on measuring optical parameters of the material (absorbance, reflectance, Raman intensity, phase shift, extinction coefficient, and others) that require complex instruments. Electrical measurements based on recording DC responses can be a simple alternative to optical measurements, particularly when the characteristics are measured in the narrow voltage range or the current flowing through the sensor is measured at one selected voltage bias. Thus, if the effect of LSPR on gas sensing can be measured by DC characteristics, it would offer unique benefits for the practical implementation of plasmonic gas sensors.

In this work, we demonstrate graphene/silicon Schottky diodes decorated with palladium nanoparticles (PdNPs) for nitrogen dioxide (NO_2_) and ammonia (NH_3_) detection. The PdNPs-decorated graphene/silicon sensor devices were illuminated with UV light of three selected wavelengths: 255, 275, and 355 nm. The PdNPs exhibit LSPR with a sharp and strong resonance absorption band close to 280 nm. Thus, the 275 nm UV LED overlaps with the plasmon resonance frequency of the PdNPs in Pd-decorated graphene and can resonantly excite the LSPR. With *I*–*V* characteristics collected for irradiated and nonirradiated sensors, we present the effects of LSPR on shifts in DC characteristics of the graphene/silicon junction under selected concentrations of oxidizing (NO_2_) and reducing (NH_3_) gases. We compare the *I*–*V* characteristics of the device obtained under UV light of 275 nm with those collected under UV light wavelengths that do not excite the LSPR in PdNPs (255 and 355 nm). The plasmon resonance phenomena dramatically enhance the sensitivity of the graphene/silicon Schottky junction sensor toward NO_2_ gas with an ultralow detection limit of 4 ppb at bias voltage, *V* = 0.3 V. Therefore, the proposed graphene/silicon Schottky junctions decorated with PdNPs are promising 2D materials for plasmon-enhanced gas-sensing applications. The investigation of the effect of LSPR excitation of Pd-decorated graphene/silicon junctions for electrical gas sensing has, to the best of the authors’ knowledge, not been reported before.

## Experimental Section

### Graphene/Silicon Diode Fabrication

Schottky diode devices were fabricated from n-type silicon and CVD-grown graphene. We used commercial n-type silicon (Si) wafers with 1–10 Ω·cm resistivity and carrier concentration between 5 × 10^14^ cm^–3^ and 5 × 10^15^ cm^–3^. First, Si wafers were cleaned, and a 90 nm SiO_2_ layer was grown on their surface by thermal oxidation. Next, the oxide layer was selectively etched to prepare windows for graphene transfer. Commercial CVD graphene grown on copper foil (Graphenea, San Sebastián, Spain) was transferred to the Si substrate using electrochemical delamination.^21,22^ Next, reactive ion etching in oxygen plasma was employed to selectively etch graphene so that part of the graphene lies directly on Si and creates a Schottky contact (G/Si junction), and some part lies on SiO_2_/Si. Then, Au/Ni (200/10 nm) electrodes were deposited on the graphene layer as-deposited on SiO_2_ using a thermal evaporator. All the lithography processes were performed by photolithography. A more detailed description of the fabrication of graphene/silicon diodes can be found elsewhere.^9^ We selected a graphene/silicon diode with an active area of 0.05 mm^2^ (width = 200 μm, length = 250 μm) for the gas-sensing exposure and sensor response measurements.

### Palladium Nanoparticle Deposition

Palladium nanoparticles (PdNPs) were fabricated and deposited onto prefabricated graphene/silicon-based Schottky diode devices. The single-step fabrication and deposition of PdNPs were made with the advanced gas deposition (AGD) technique (Ultra Fine Particle Equipment, ULVAC Ltd., Japan)^23^ from its pure metallic precursor (purity, 99.95%). Palladium metal pellets placed in a graphite crucible were positioned in the axial center of an induction copper coil in the lower evaporation chamber of the AGD compartment at vacuum pressure, *P*_1_ *=* 86 Torr. The Schottky diode devices were loaded onto a movable substrate holder in the upper deposition chamber of the second AGD compartment, held at vacuum pressure, *P*_2_ = 1.0 Torr. The two AGD chambers are connected via a 3 mm diameter transfer pipe and pre-evacuated to a vacuum pressure of 10^–2^ Torr. The metal precursor was then inductively heated to its melting temperature at a power of 2.2 kW, and simultaneously, the metal vapor was cooled in high-purity helium (He) gas flow of 30 L/min as introduced into the evaporation chamber. The metal atoms ejected from the precursor melt rapidly lose their energy in collision with gas atoms, leading to the spontaneous nucleation and growth of log-normal distributed nanoparticles.^20^ The as-produced nanoparticles are accelerated and transferred to the upper chamber, where the deposition of nanoparticles takes place onto the Schottky devices while the XYZ stage motor moved at a speed of 2.5 mm/s. The fabrication parameters, including the power, pressure, deposition cycles, and speed of the substrate holder stage, were optimized to deposit highly dispersed PdNPs onto the graphene/silicon sensing layer.^24^ The fabrication and deposition parameters of the AGD process were optimized to produce noncontacting monodispersed PdNPs with a log-normal size distribution and surface coverage close to the percolation threshold. The NP’s surface density was indirectly evaluated from a high-precision *I*–*V* measurement of a device as-deposited with PdNPs, where the NP’s distribution across the interdigitated electrodes has a direct effect on the measured drain current.

The surface morphology and structure of the as-deposited PdNPs were analyzed by scanning electron microscopy (SEM) employing a Zeiss LEO 1550 instrument. The optical property of the as-fabricated PdNPs was measured by a double-beam PerkinElmer Lambda 900 spectrophotometer equipped with a BaSO_4_-coated integrating sphere. The PdNPs show strong and sharp absorption peaks in the UV region, indicating the localized surface plasmon resonance phenomenon characteristic of dispersed PdNPs with a narrow size distribution.^25^ Figure 1a shows a high-resolution SEM image of the as-deposited PdNPs and depicts the deposition of highly monodispersed nanoparticles with an average particle size of 3 ± 0.7 nm. The UV–visible spectrum shown in Figure 1b presents the sharp absorption band due to the LSPR of PdNPs with the maximum absorbance at 283 nm (resonance wavelength λ_res_ marked on the graph).

**Figure 1:**
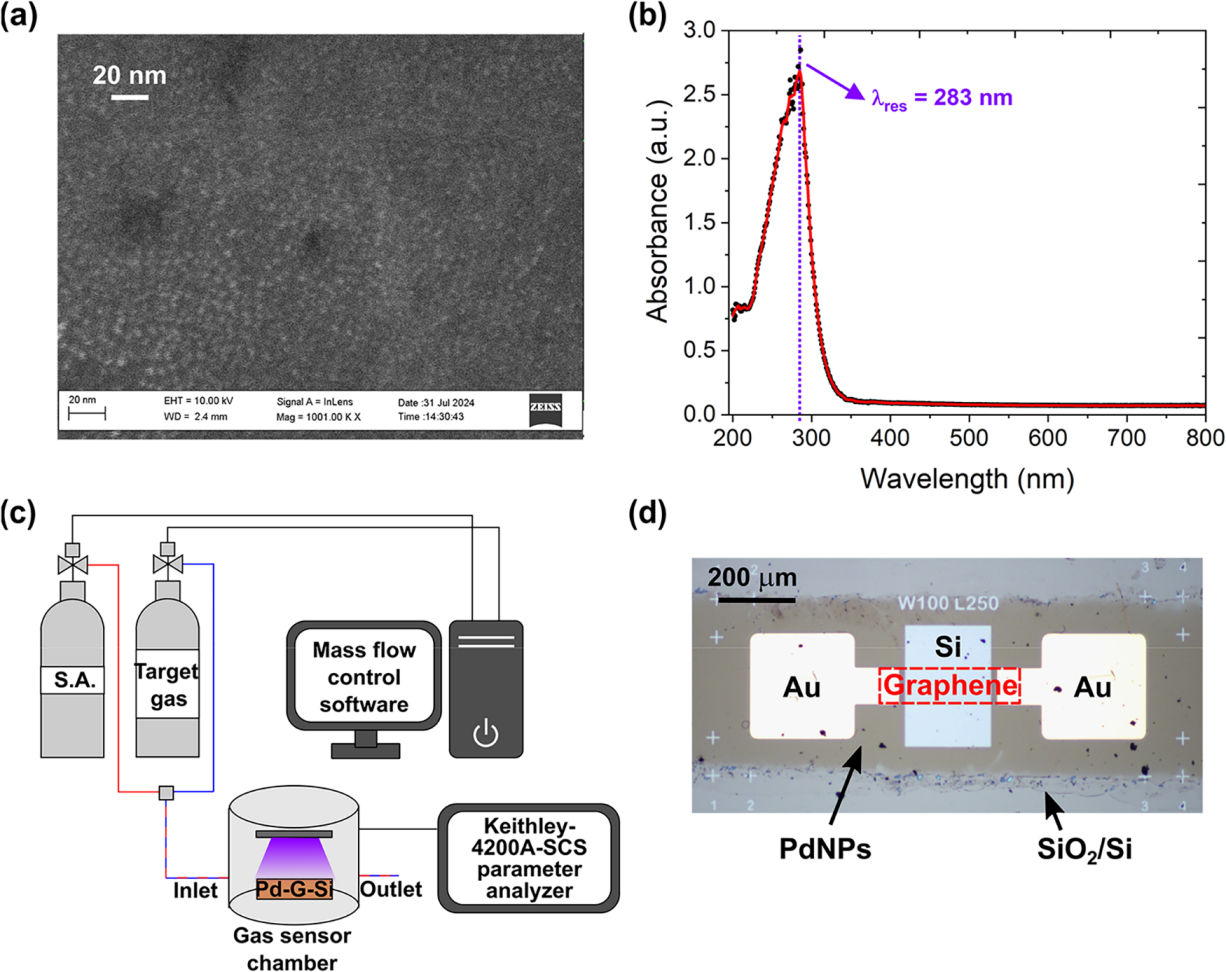
(a) SEM image of the PdNPs. (b) Absorbance spectrum of PdNPs showing the sharp plasmon resonance at λ_res_ = 283 nm. The spectrum was measured for PdNPs deposited on quartz substrates employing conditions identical to those used for the G/Si diode sensors. (c) Illustration of the experimental setup for measuring gas-sensing responses of the irradiated G/Si Schottky diodes decorated with PdNPs (Pd-G/Si). The target gas is NO_2_ or NH_3_. (d) Optical microscopy image of the graphene/Si Schottky junction sensor showing two Schottky junctions (left and right). The SiO_2_/Si substrate and regions decorated with PdNPs are marked with arrows. The region of the graphene layer placement is indicated with a red dashed rectangle.

### DC Characteristic Measurements

DC response measurements were performed in an MPS150 probe station (FormFactor). Titanium needles were used to connect the investigated Schottky diode with measuring and biasing units. A Keithley-4200A-SCS parameter analyzer with two medium power source-measure units (type 4201-SMU) was used for all electrical measurements. One contact was connected to graphene (through Au/Ni electrode), while another was connected to the silicon (etched and metalized backside of the Si wafer). Here, the Au/Ni makes ohmic contact with the graphene, while the graphene/silicon (G/Si) interface forms a Schottky barrier that exhibits rectifying behavior. Current–voltage (*I–V*) characteristics were collected for the Schottky diode, where negative voltages represent the reverse bias, and positive voltages represent the forward bias for the G/Si junction. Each *I–V* characteristic was measured from 0 to +2 V or 0 to −2 V with a 5 mV step and combined in one graph. Measurements in this limited voltage range reduced the detrimental effect of discharging deep levels in n-doped Si, which otherwise can induce shifts of the recorded *I–V* characteristics. The investigated Schottky diode device was kept in a metal gas chamber of ∼120 cm^3^ volume with a gas inlet and outlet to maintain the selected gas atmosphere surrounding the sensor. UV LEDs with selected wavelengths were employed for light-assisted gas-sensing measurements. Their distance to the sensing surface was adjusted to obtain the same optical power density of 2 mW/cm^2^. We applied three different UV LEDs with wavelengths of 255 nm (Inolux, type IN-C35PPCTGU0), 275 nm (ProLight Opto, type PB2D-1CLA-TC), and 355 nm (Roithner LaserTechnik, type XSL-355–3E-R6), respectively. The UV LEDs were selected to investigate the effect of light irradiation on the DC characteristic close to the LSPR peak of the PdNPs at 283 nm. The 275 nm LED closely matches the measured LSPR. At 255 nm, the plasmon peak is about half of the maximum intensity. At 355 nm, the absorption associated with the plasmon resonance is minimal. UV light shifts the zero-bias current point in the *I–V* characteristic by around hundreds of mV due to the photovoltage effect. The repeatability of the electrical properties of the junction was monitored each day by measuring the reference *I–V* characteristic in laboratory air (relative humidity RH of ∼30–40%) in the dark before initializing sensing experiments to control any possible aging of the sensor and drift in the baseline parameters.

### Gas-Sensing Experiments

Nitrogen dioxide (NO_2_) and ammonia (NH_3_) were used as target gases for gas-sensing experiments due to their opposite redox properties. NO_2_ is an oxidizing molecule (strong electron acceptor), and NH_3_ is a reducing agent (weak electron donor due to the lone pair on nitrogen). Both gases are important industrial gases, but also air pollutants harmful to humans and the environment.^26^ Dry synthetic air (S.A.) was used as a carrier gas and reference atmosphere in our studies. We mixed S.A. with target gases at specific proportions to obtain selected concentrations: 1–7 ppm of NO_2_ and 5–15 ppm of NH_3_. The gas flow was set to 50 mL/min (regulated by mass flow controllers – Analyt-MTC, model GFC17) and maintained during all gas-sensing experiments. The carrier gas (in the dark or during UV irradiation) was admitted for ∼1 h before starting designated sensing experiments to stabilize the baseline properties of the sensor and reduce the drift. All sensing experiments were conducted at room temperature (RT ∼25 °C) and ambient pressure (∼1 bar). The gas-sensing responses of the G/Si diode demonstrated in this work are presented as the relative change in the current flowing through the sensor, *I*_S_, in the target gas and the current, *I*_0_, measured in dry synthetic air (S.A.) at a specific voltage bias: (*I*_S_–*I*_0_)/*I*_0_. Gas sensing experiments with G/Si Schottky diodes were realized using the setup illustrated in Figure 1c. The optical microscopy image of the exemplary G/Si diode decorated with PdNPs is demonstrated in Figure 1d.

## Results and Discussion

Figure 2 shows the *I–V* curve responses when the Pd-G/Si device was exposed to NO_2_ gas (1–7 ppm). The current *I* is depicted as the absolute value in all figures. The region of the *I–V* characteristic mostly affected by the target gas in the dark is near the curve bending for positive voltages between 0.2 and 0.6 V (Figure 2a). The reverse part of the *I–V* characteristic is barely affected, and the shift of the zero-bias current point is random and not correlated with the gas concentration and can be explained by the lower *I*-values of at least 1 order of magnitude smaller when compared with UV-light irradiated experiments. We claim that some tiny, nonzero current values at zero voltage are induced by recharging of deep levels during the voltage scan. When the sample is irradiated, the current is much higher, and this detrimental effect can be omitted. Measurements of higher currents are also less affected by noise, and hence the sensing responses are more stable. Notably, to realize stable gas detection by simple electronics, voltage bias should be adjusted within the gas sensitivity region and at a current level >1 nA. UV light shifts all the characteristics toward higher positive voltages due to the photovoltage effect and increases the reverse current. For the longest wavelength (355 nm), the changes induced by NO_2_ are visible for reverse voltages and close to the bending region of the *I–V* characteristics (Figure 2b). The same regions are affected by the shortest, 255 nm wavelength (Figure 2d). The voltage shift and increase of current near the zero-bias current point are most pronounced at 275 nm UV irradiation close to the LSPR of the PdNPs (Figure 2c). This is attributed to a combination of the electromagnetic near-field enhancement that promotes electron–hole pair excitation in the supporting graphene/Si and interfacial charge transfer, whereby hot electrons from the metal can overcome the Schottky barrier and enter the graphene conduction band. Figure 3 presents the analogous *I–V* characteristics measured at different concentrations of NH_3_. Under NO_2_ exposure, the *I–V* curves shift to higher positive voltages due to the photovoltage effect upon UV irradiation, while the reverse current remains approximately unchanged. NH_3_ clearly induces smaller changes in the *I–V* characteristics than NO_2_, both near the curve bending and in the reverse bias regime.

**Figure 2:**
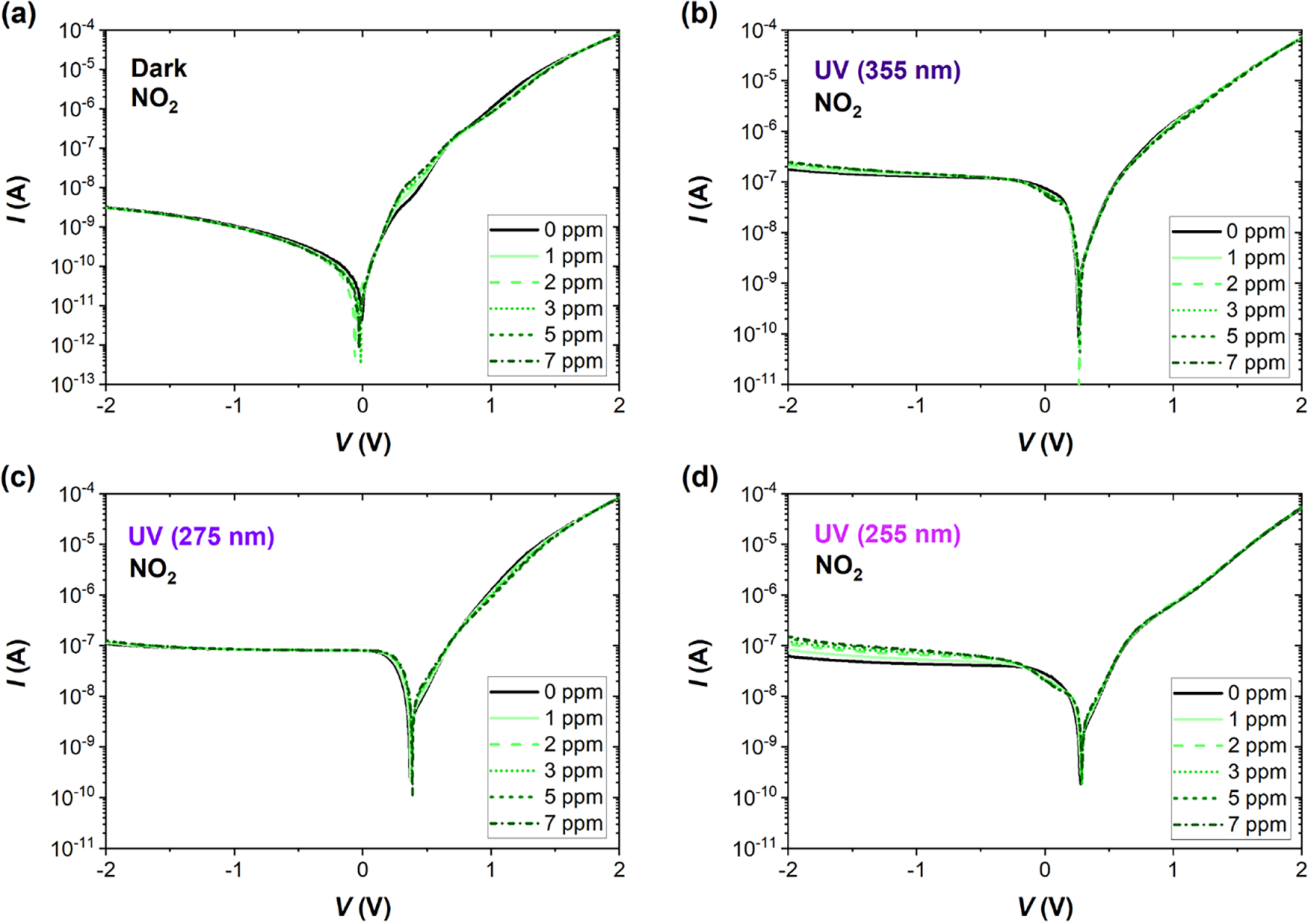
Current–voltage (*I–V*) characteristics of the G/Si sensor in S.A. and selected concentrations of NO_2_ (1–7 ppm) (a) in the dark and under UV irradiation of (b) 355 nm, (c) 275 nm, and (d) 255 nm wavelength. The instability of the zero-bias current point in the dark can be explained by the low measured current values (less than 10^–12^ A) and the detrimental effect of deep levels recharging during the voltage scan.

**Figure 3:**
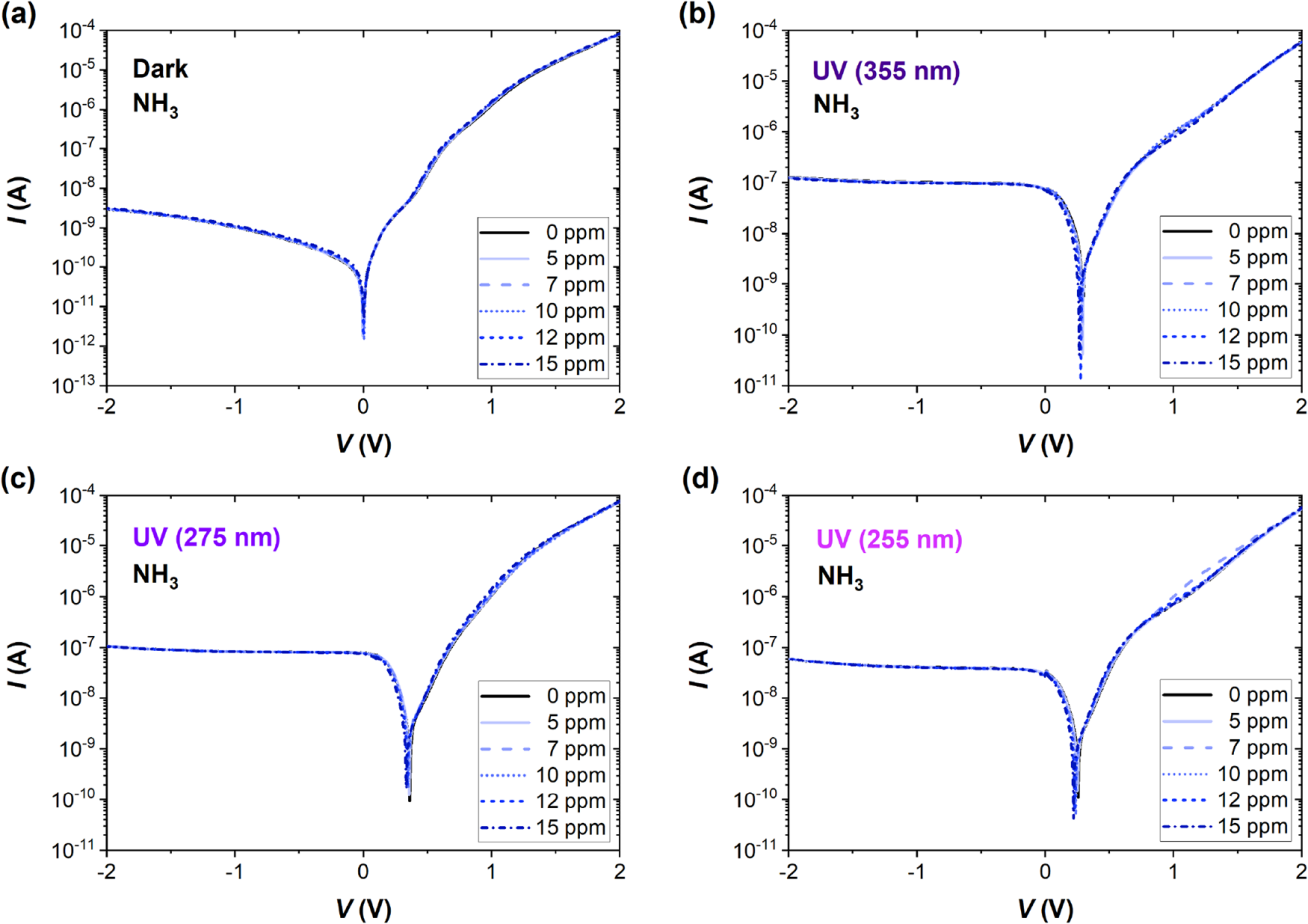
Current–voltage (*I–V*) characteristics of the G/Si sensor in S.A. and selected concentrations of NH_3_ (5–15 ppm) (a) in the dark and under UV irradiation of (b) 355 nm, (c) 275 nm, and (d) 255 nm wavelength. The instability of the zero-bias current point in the dark can be explained by the low measured current values (less than 10^–12^ A) and the detrimental effect of deep levels recharging during the voltage scan.

Figure 4 shows *I–V* plots within the 0.1 to 0.5 V region of the device’s *I–V* characteristics in Figures 2 and 3. Comparing the results obtained under UV irradiation for NO_2_ (1–7 ppm) and NH_3_ (5–15 ppm) gases, a clear dependence is evident between the position of the zero-bias current point and the concentration of both target gases. The arrows in the graphs point the direction of changes during exposure to increasing concentrations of NO_2_ and NH_3_ gases. The sensor response results show an opposite behavior for oxidizing and reducing gases, and the shift increases with the increasing concentration for all applied UV LEDs. At a selected voltage bias, the adsorption of NO_2_ onto the sensing layer increases the current flowing through the Pd-decorated G/Si junction, while sensors exposed to NH_3_ gas lead to a decrease in the current across the junction. This is attributed to the electrophilic and nucleophilic properties of NO_2_ and NH_3_ gases, respectively. NO_2_, being a strong electron acceptor, depletes the charge density from the conduction band. Due to its electron-donating properties, NH_3_ induces the opposite effect and injects additional charge carriers into the conduction band. Therefore, we see a shift in the *I–V* curve to the opposite direction for each target gas, making them easier to distinguish.

**Figure 4:**
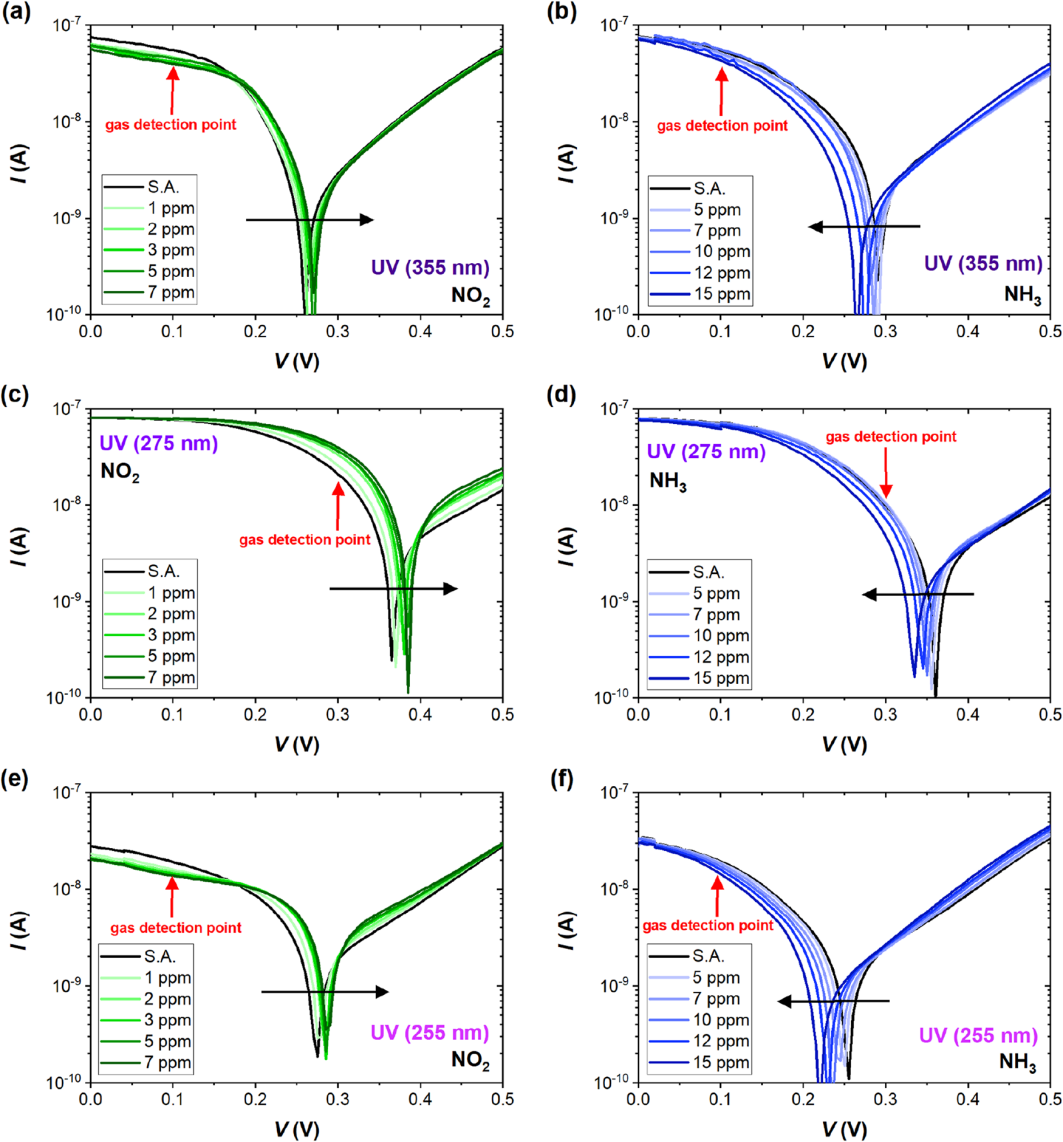
A close-up of the current–voltage characteristics of the G/Si sensor in S.A. and selected concentrations of NO_2_ (1–7 ppm) and NH_3_ (5–15 ppm) under UV irradiation of 355 nm (a, b), 275 nm (c, d), and 255 nm (e, f) wavelength with black arrows marking the direction of shift near the bending of the *I–V* curves. Red arrows mark the “gas detection point” considered in further sensor response analysis (*V* = 0.1 V for UV 355 and 255 nm, and *V* = 0.3 V for UV 275 nm).

The measurements performed for UV-irradiated (275 nm) G/Si diodes without PdNPs showed that the current responses at a fixed voltage bias of 0.2–0.3 V are lower compared to the Pd-decorated sensor exhibiting plasmon resonance effects (see Figure S1). For example, at *V* = 0.3 V, the current response to 1 ppm of NO_2_ is 28% for the nondecorated G/Si and 53% for the Pd-decorated G/Si sensor device. Detailed results for nondecorated G/Si Schottky junction devices are demonstrated in our previous work.^27^

PdNPs are also known to possess a high affinity to hydrogen, so we have performed additional experiments with 50 ppm of H_2_ for the G/Si sensor operating in the dark and LSPR conditions (UV light of 275 nm). Supporting Figure S2 shows that the electrical response to 50 ppm of H_2_ does not exceed that obtained for NO_2_ or NH_3_. The voltage shift of the *I–V* characteristic is only 5 mV under UV light, and the current response (*I*_S_ *–I*_0_)/*I*_0_ is minor.

The forward bias regime in the G/Si Schottky diode consists of an exponential part of the *I–V* curve associated with the G/Si interface and a linear part for higher positive voltages where graphene resistance dominates. The transition part of the *I–V* curve between the two regions occurs between 0.2 and 0.6 V, and this part is characteristic for graphene Schottky diodes. As reported before, this part can be susceptible to the induced potential near the graphene surface by adsorbed molecular dipoles (gating effect).^27^ However, it does not distinguish between oxidizing and reducing species since the change is in the same direction regardless of the gas. Our results show that the effect of the gating potential is visible mainly for NO_2_ sensing in the dark (see Figure 2a and region of voltages between 0.2 and 0.6 V) and becomes weaker under UV irradiation. The exponential part near the zero-bias current point and the reverse regime are mostly affected by changes in the gas surrounding the sensor. We want to highlight that the region of interest for gas sensing (reverse voltage bias and part of the *I–V* characteristic near the zero-bias current point) is reproducible for Schottky diodes of the same dimensions fabricated within the same batch (see Figure S3).

Figure 5 summarizes the calibration curves derived as changes in the current flowing through the sensor ((*I*_S_ *–I*_0_)/*I*_0_ responses) for NO_2_ or NH_3_ concentrations and shows the relative voltage shift (*V*_S_ *–V*_0_)/*V*_0_ derived as the lateral shift of the recorded *I–V* curves at the specific current level, i.e., 10 nA, with reference to S.A. conditions (*V*_0_). The voltage shift depends on the particular UV LED applied, but (*V*_S_ *–V*_0_)/*V*_0_ values are generally lower than (*I*_S_ *–I*_0_)/*I*_0_ responses (one magnitude lower in the case of NO_2_). Voltage shift is observed to saturate for higher NO_2_ concentrations, which is a common observation for this gas, as electrophilic molecules possess a strong affinity to graphene and Pd, and rapidly occupy surface binding sites with charge transfer. Therefore, at higher concentrations in the ppm range, the electrical responses may saturate earlier than for other gases. Table 1 summarizes the Pd-G/Si sensor responses for NO_2_ and NH_3_ of the same concentration (7 ppm) under selected UV-light irradiation conditions and two voltage biases–in the reverse voltage bias (*V* = −2 V) and near the zero-bias current point, which corresponds to voltage of 0.1 or 0.3 V depending on the applied UV LED (designated as “@gas detection point”). The responses (*I*_S_ *–I*_0_)/*I*_0_ are clearly higher for NO_2_ than NH_3_ in all cases. Excess holes are further generated due to NO_2_ adsorption and the electron–hole recombination at the interface enhances the flow of current across the interface. Upon NO_2_ exposure, the reverse regime becomes more sensitive to NO_2_ adsorption when UV light does not correlate with the resonance wavelength. Notably, employing the shortest UV wavelength (255 nm) resulted in a current response of 142% for 7 ppm of NO_2_ and a theoretical detection limit (DL) of 120 ppb. Illuminating the devices with 355 nm wavelength increased the current in the reverse voltage bias; however, the (*I*_S_ *–I*_0_)/*I*_0_ response was three times lower compared with 255 nm at the same NO_2_ concentration. The change in the bending region of the device’s *I–V* curve becomes larger when the plasmon resonance is excited. DL obtained under plasmonic conditions (UV 275 nm) at *V* = 0.3 V is as low as 4 ppb, showing the superiority of NO_2_ sensing in such operating conditions. DL achieved in this work is significantly lower compared to the previous reports for NO_2_ sensors utilizing plasmonic nanoparticles; e.g., DL of 250 ppb was obtained for Au-decorated ZnO irradiated with green light.^28^ Reports on graphene decorated with PdNPs exhibited DL values of 1.2 ppm for NO_2_ and 5 ppm for NH_3_ at 150 °C.^29^ The same sensors exhibited response times of ∼5 min and recovery times of ∼35 min. Other sensors that utilized graphene and AuNPs exhibited response and recovery times between 12 and 17 min for 1 ppm of NO_2_ measured by UV–visible spectroscopy.^30^ Although longer incident wavelengths generally prolong the response and recovery time of the sensor, the Pd-decorated G/Si sensors show relatively fast recovery. Thus, the sensors’ response and recovery times for NO_2_ gas were measured to be 300 and 400 s, respectively. In contrast, we propose measuring sensing responses only by the device’s DC characteristics, while other plasmonic sensors usually rely on measuring changes in optical properties of the sensing material (plasmonic structures), such as absorbance, reflectance or Raman intensity, which requires more complex apparatus.^31,32^

**Figure 5:**
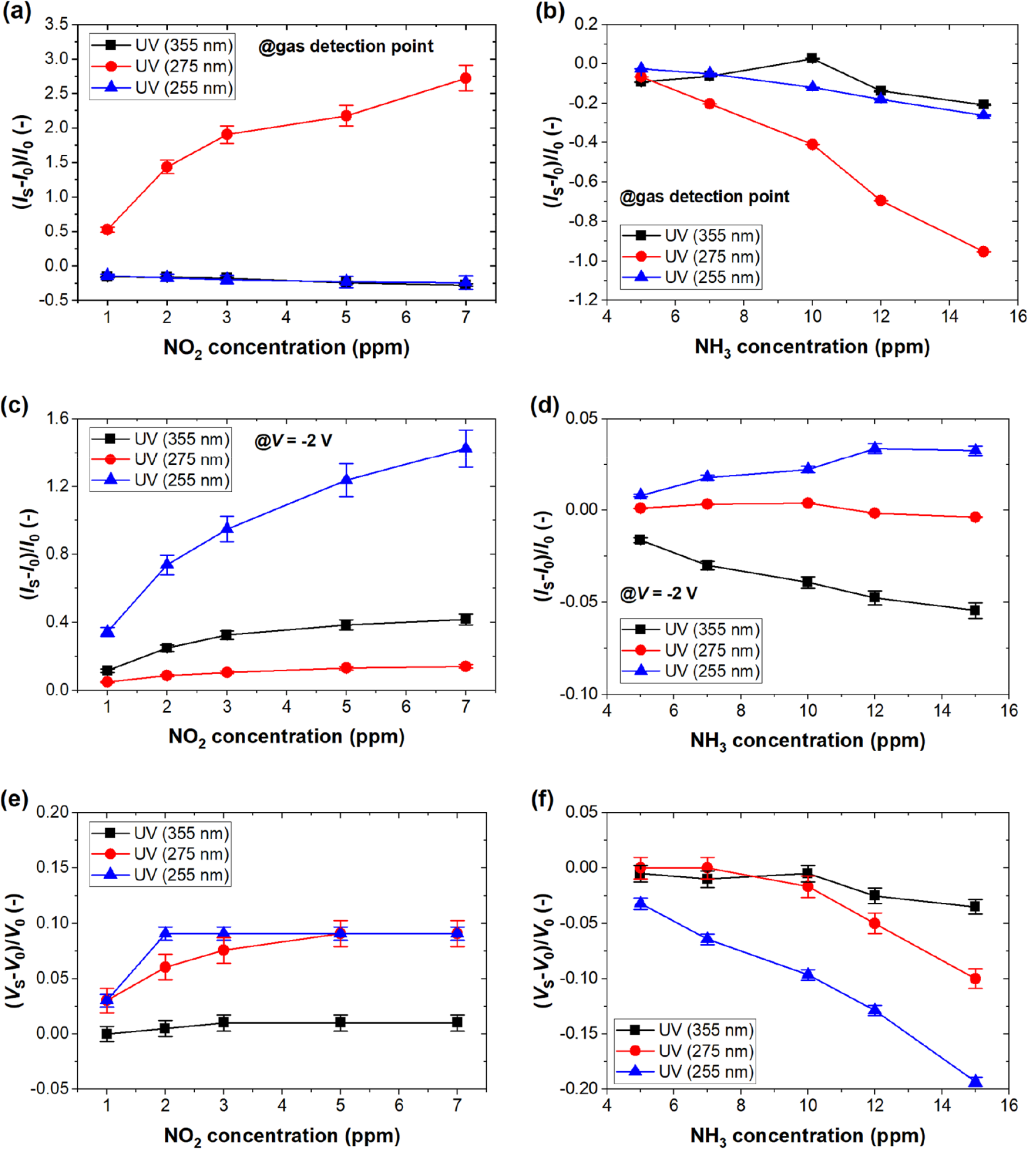
Effect of UV light on NO_2_ and NH_3_ sensing responses illustrated by the relative change in the current flowing through the Pd-G/Si sensor at gas detection point at the voltage bias of 0.1 V, 0.3, and 0.1 V for UV light of 355, 275, and 255 nm wavelength (a, b); the relative change in the current flowing through the sensor at *V* = −2 V (c, d); the shift of *V*_S_ in reference to *V*_0_ in S.A. at the current level of 10 nA (e, f).

**Table 1:** Comparison of Current Responses (*I*_S_–*I*_0_)/*I*_0_ Obtained for 7 ppm of NO_2_ and NH_3_ for the G/Si Sensor Decorated with PdNPs under Selected UV Light Conditions and Different Voltage Biases

	NO_2_ (7 ppm)		NH_3_ (7 ppm)	
	@*V* = −2 V	@gas detection point	@*V* = −2 V	@gas^a^ detection point
dark	–1%	140%	<1%	–1%
UV 355 nm	42%	–28%	–3%	–6%
UV 275 nm	14%	**272%**	<1%	**–20%**
UV 255 nm	**142%**	–24%	2%	–5%

a The bending region of the *I–V* curve of the high sensitivity to adsorption of target gas occurs at different voltages, depending on the ambient light–the voltage bias corresponding to the gas detection point is 0.3 V in the dark, 0.1 V for UV 355 nm, 0.3 V for UV 275 nm, and 0.1 V for UV 255 nm (as marked with red arrows in Figure 4).

Regarding the effect of humidity on the responsivity of the Schottky diode device from our previous studies, the relative sensor response of pristine G/Si Schottky diode sensors was lower for NO_2_ (1–7 ppm) and NH_3_ (5–10 ppm) gases with 40% relative humidity and under UV light (275 nm) irradiation.^27^ Therefore, we expect a similar trend for the Pd-decorated G/Si Schottky devices.

Considering the effect of gas mixture on the device responsiveness, we performed experiments with mixed concentrations of NO_2_ (5 ppm) and NH_3_ (5 ppm) under a UV-irradiated sensor (plasmonic resonant wavelength of 275 nm). We observed that the zero-bias current point almost coincides with that for pure NH_3_ (see Supporting Figure S4). A recently reported paper about sensing mixed concentrations of NO_2_ and NH_3_ by a WS_2_-based sensor revealed that, depending on the exposure time of both gases and their concentrations, the sensor’s response can be dominated by one of them (NO_2_ dominates the response in shorter exposure, whereas NH_3_ slowly dominates the sensor’s resistance in longer exposure time).^33^ Therefore, the DC responses are not completely canceled out by target gases of opposite redox properties. We expect that a similar effect may occur for graphene-based Schottky diodes which should be confirmed by further studies.

We expect that the choice of UV light wavelength can also affect the gas adsorption/desorption kinetics and the repeatability of sensing responses. Figure S5 shows transient sensor response curves for five cycles of NO_2_ (3 ppm) or NH_3_ (10 ppm) under UV light of 275 or 255 nm wavelength, recorded at a fixed voltage bias in the exponential part of the *I–V* characteristics. The UV irradiation in the LSPR (UV 275 nm) wavelength yields reversible and repeatable sensor responses to NO_2_ gas. The shorter wavelength (UV 255 nm) does not lead to complete recovery after the first detection cycle. Time-resolved measurements indicate the limitation of utilizing the G/Si Schottky diode sensors for NH_3_ gas sensing. Notably, none of the UV LEDs employed in this work facilitated sensor recovery after exposure to NH_3_ gas within 20 min, and hence, the baseline continuously drifts as a function of cycling time. This drift was also visible in *I–V* characteristics and it shifted slowly to the left during longer measurement cycles, even in only S.A. However, recovery of the *I–V* characteristics was obtained overnight when the sensor was stored in the dark in laboratory air, suggesting a prolonged sensor recovery process after NH_3_ adsorption and slow surface NH_3_ reaction kinetics rather than electronic effects.

The recorded *I–V* characteristics of the Schottky diode sensor indicate a few separate regions of gas sensitivity for which the mechanisms of detection can be described by different processes. The voltage shift visible in the reverse bias regime (negative voltages) is associated with the changes in the properties of the G/Si junction, particularly the Schottky barrier height. The current flowing through the junction, *I*_S_, during UV-light irradiation can be expressed by the equation^6^

1 $$I_{S}=I_{\mathrm{sat}}\left(e^{\mathrm{eV}/\eta \mathrm{kT}}-1\right)-I_{\mathrm{ph}}$$

where *I*_sat_ represents the saturation current, *e* is the electronic charge, *V* is the applied voltage, η is the ideality factor, *k* is the Boltzmann constant, *T* is the absolute temperature, and *I*_ph_ is the photocurrent. The photocurrent *I*_ph_ is 2 orders of magnitude larger than the saturation current *I*_sat_ (at *V* = −2 V, *I*_sat_ = 3.2 × 10^–9^ A, and *I*_ph_ = 1.1 × 10^–7^ A in S.A. under UV (275 nm) light). The Schottky barrier height (*e*ϕ_B_) can be calculated from the saturation current defined as

2 $$I_{\mathrm{sat}}=AA^{*}T^{2}e^{-\left(e\phi_{B}/\mathrm{kT}\right)}$$

where *A* is the Schottky barrier area (0.05 mm^2^), A* is the Richardson’s constant (112 A·cm^–2^·K^–2^ for n-type Si), and ϕ_B_ is the Schottky barrier potential. The ideality factor η calculated from the *I–V* curve for the sensor in S.A. irradiated with UV light (275 nm) is 5.72, which yields *e*ϕ_B_ = 0.62 eV, which is consistent with previous reports on graphene/Si diodes.^9,17^

We assume that ambient gases change the Schottky barrier height and induce the voltage shift of the *I–V* characteristic. Therefore, for two different gases (e.g., target gas 1 = NO_2_ and reference gas 2 = S.A.), the current flowing through the G/Si junction can be written as

3 $$I_{1}=AA^{*}T^{2}e^{-\left(e\phi_{B1}/\mathrm{kT}\right)}e^{eV_{1}/\eta \mathrm{kT}}-{AA^{*}T^{2}e^{-\left(e\phi_{B1}/\mathrm{kT}\right)}-I}_{\mathrm{ph}}$$

4 $$I_{2}=AA^{*}T^{2}e^{-\left(e\phi_{B2}/\mathrm{kT}\right)}e^{eV_{2}/\eta \mathrm{kT}}-{AA^{*}T^{2}e^{-\left(e\phi_{B2}/\mathrm{kT}\right)}-I}_{\mathrm{ph}}$$

The second term is negligibly small and can be omitted. Also, we can assume that the photocurrent *I*_ph_ is the same in both equations. Then, for the zero current conditions (*I*_1_ = *I*_2_ = 0), the equations are simplified to

5 $$I_{\mathrm{ph}}=AA^{*}T^{2}e^{-\left(e\phi_{B1}/\mathrm{kT}\right)}e^{eV_{1}/\eta \mathrm{kT}}$$

6 $$I_{\mathrm{ph}}=AA^{*}T^{2}e^{-\left(e\phi_{B2}/\mathrm{kT}\right)}e^{eV_{2}/\eta \mathrm{kT}}$$

By dividing eqs 5 and 6 and rearranging the variables

7 $$1=e^{-\left(e\phi_{B1}-e\phi_{B2}/\mathrm{kT}\right)}e^{eV_{1}-eV_{2}/\eta \mathrm{kT}}$$

8 $$\phi_{B2}-\phi_{B1}=\frac{1}{\eta }\left(V_{2}-V_{1}\right)$$

9 $${\Delta \phi }_{B}=\frac{1}{\eta }\Delta V$$

Adsorption of gases and charge transfer between the sensor surface and gas molecules can increase (NO_2_) or decrease (NH_3_) *e*ϕ_B_. Thus, the voltage shift represents the changes in the Schottky barrier height, which can be found from the zero voltage shift (the shift of the point in the *I–V* curve where the current is minimal). For example, under UV (275 nm), the change in the Schottky barrier height *e*Δϕ_B_ is between 0.9 and 3.5 meV for NO_2_ concentrations between 1 and 7 ppm and between −0.9 and −4.4 meV for NH_3_ concentrations between 5 and 15 ppm, respectively.

Figure 6 depicts the band energy diagrams of the G/n-Si junction device at equilibrium zero bias, forward bias, and reverse bias (panel (a)). We consider our graphene layer to have defects and behave slightly p-doped due to oxygen-containing groups in the ambient air atmosphere. The Schottky barrier for the G/Si junction behaves according to the applied bias voltage, where an applied reverse bias tends to push the Fermi level of the graphene upward, injecting electrons over the Si conduction band, thereby lowering the Schottky barrier. Upon exposure to NO_2_ and NH_3_, the *e*ϕ_B_ increases or decreases, as NO_2_ acts as a p-type dopant and NH_3_ as an n-type dopant to graphene (panel (b)), respectively. The behavior of the Pd-decorated G/Si junction upon illumination with a wavelength corresponding to the LSPR illustrated the change in the Fermi level of graphene, which is modulated by the forward and reverse bias under UV illumination (panel (c)). This effect becomes enhanced when a highly electrophilic NO_2_ gas extracts the majority charge carriers from the channel. Thus, when a reverse bias is applied, the Pd-decorated graphene opens more accessible states for the photogenerated electrons, resulting in an increase in the charge carrier density across the channel. When graphene is exposed to highly reducing gases, extra electrons are injected, causing an upshift in the Fermi level toward the Si conduction band, lowering the Schottky barrier height. However, the adsorption of highly oxidizing gases, such as NO_2_, induces additional holes, leading to an increase in the Schottky barrier height due to the down-shift in the Fermi level toward the valence band of Si. Thus, the injection of majority carriers from the graphene to the n-Si is determined by the Schottky barrier height, and irradiation of the G/n-Si with 275 nm further modulates the current flow across the junction.

**Figure 6:**
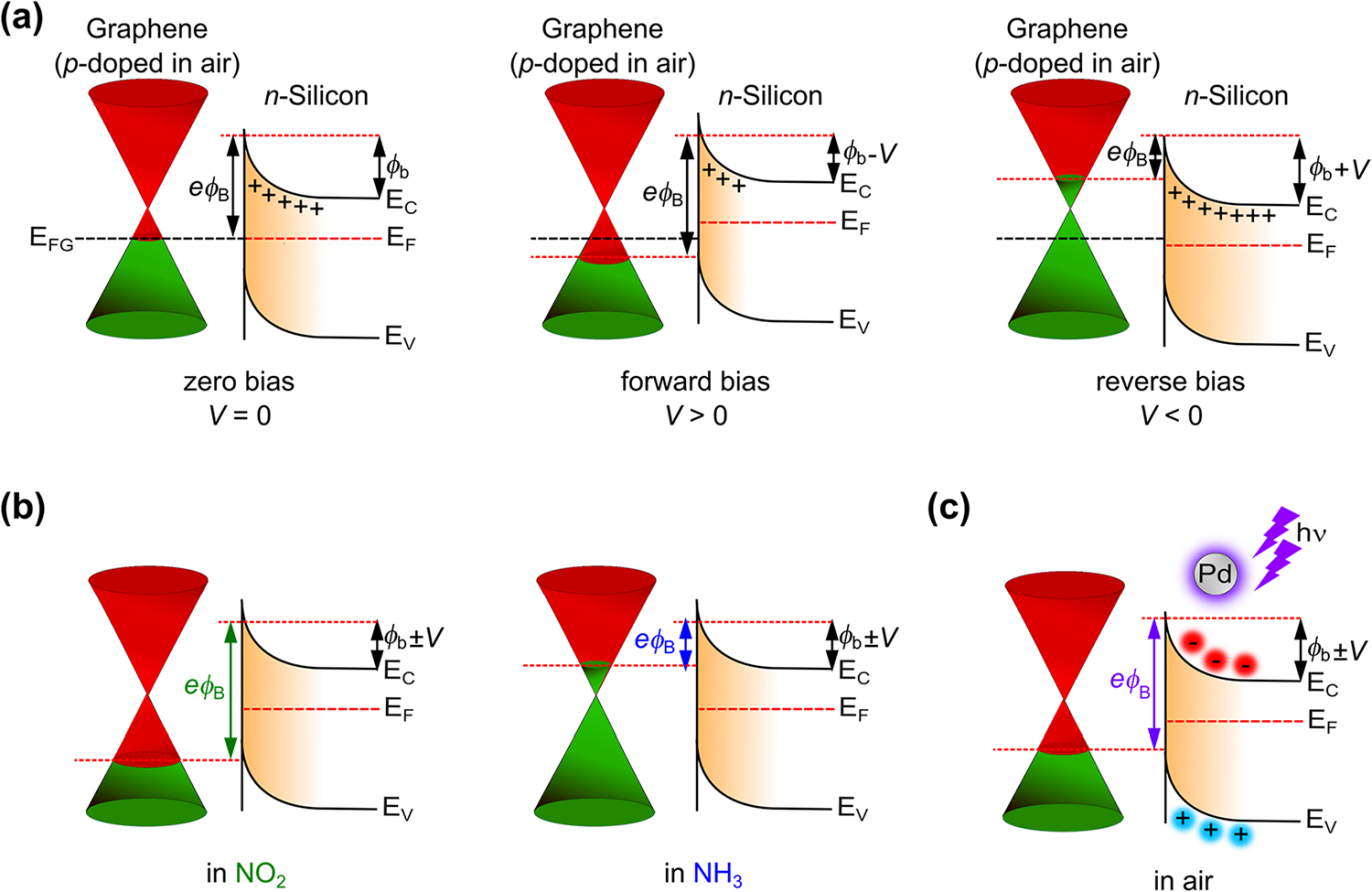
Energy band diagrams for G/n-Si junctions at (a) different external bias voltages, (b) in NO_2_ (p-doping agent) and NH_3_ (n-doping agent), and (c) after Pd decoration and under illumination with UV light. *e*ϕ_B_, ϕ_b_, and *V* denotes the Schottky barrier height, the built-in potential at the interface, and the external bias voltage applied during the device operation, respectively. *E*_C_, *E*_V_, *E*_FG_, and *E*_F_ denote the conduction, valence, and Fermi level energy bands for graphene and silicon, respectively.

## Conclusions

A graphene/silicon Schottky junction (G/Si) device was fabricated, and plasmonic palladium nanoparticles (PdNPs) were subsequently deposited onto the sensing layer for UV-light-enhanced NO_2_ and NH_3_ gas detection. The gas-sensing property of PdNPs-decorated G/Si Schottky diode sensors was investigated from the device’s current–voltage (*I–V*) measurements under dark conditions and UV-light irradiations at selected wavelengths. The measured *I–V* curve of the device under gas exposures indicates a shift in bias voltage characteristic to the wavelength of the UV light and exhibits distinctive sensor sensitivity toward each gas under test (NO_2_ and NH_3_).

The effect of UV irradiation on the Pd-decorated graphene sensor depends on the wavelength, with a maximum coinciding with the PdNPs plasmon absorption. Notably, the incident light wavelength, which aligns with the localized surface plasmon resonance (LSPR) property of the PdNPs, provided a significant sensor response close to the zero-bias current point of the *I–V* characteristic. The highest sensitivity of the sensor was therefore obtained for NO_2_ gas at *V* = 0.3 V, where the *I–V* characteristics of the device were shifted gradually to higher positive voltages in relation to the increasing NO_2_ gas concentration. The sensor theoretical detection limit calculated for such conditions is in the subppb range of 4 ppb for NO_2_ gas. The current response to NO_2_ gas was almost 14 times higher than to NH_3_ of the same concentration (7 ppm) when a UV LED of 275 nm and a voltage bias of 0.3 V were employed. At the same time, even though the sensor sensitivity to NO_2_ gas was higher than to NH_3_ under UV light illumination and external voltage biasing conditions, the selectivity factor was lower in the dark and under UV light of wavelengths that do not induce LSPR in PdNPs (255 and 355 nm). The plasmon resonance in the PdNPs affects the carrier transfer and charge redistribution at the graphene surface when the gas molecules are adsorbed. LSPR strongly affects the Schottky barrier at the graphene/silicon interface, which is further reflected by the diode threshold voltage shift due to the NO_2_ gas adsorption. Thus, the graphene sensing layer decorated with PdNPs that exhibit LSPR in the UV spectral range combines the graphene photon activation and plasmonic effect for highly sensitive and selective gas sensors. Both effects are expected to influence the gas-sensing properties of graphene/silicon Schottky junctions.

Furthermore, we present a very convenient method of investigating the signal responses of plasmonic gas sensors based on the device’s DC characteristics measurements, which is simplified compared to optical and spectroscopic methods. Even though the photoinduced graphene-based Schottky diodes for gas sensing were reported before,^34^ we focused on plasmon-enhanced detection characterized only by DC responses. In contrast to optical sensors, we demonstrated a miniaturized sensing device of low energy consumption associated only with polarizing the Schottky junction and the UV LED that induces the LSPR effect in Pd-decorated graphene devices. The results suggest that the most sensitive part of the sensor is limited to the graphene area where it forms the junction with n-doped Si. It means that only this part requires decoration by PdNPs and suggests a reduction of the applied PdNPs and the costs of sensors preparation. Therefore, our findings confirm that the design and fabrication of 2D materials decorated with plasmonic metal nanoparticles with the application of UV-light irradiation at a particular wavelength present a promising gas-sensing system for detecting and analyzing both oxidizing and reducing gases.

## Supplementary Material



## Notes

Current–voltage characteristics of the nondecorated G/Si sensor in NO_2_ and NH_3_ under UV light (275 nm); current–voltage characteristics of the Pd-G/Si sensor in H_2_; repeatability of characteristics of the Pd-G/Si sensors; Pd-G/Si sensor response to mixed concentrations of NO_2_ and NH_3_; time-domain responses of the Pd-G/Si sensor under UV light (275 and 255 nm) (PDF)
