# Supplementary material for: Plasmon-Induced Graphene/Silicon Schottky Junctions for Ultrasensitive Gas Sensing
Source: ACS Sens. 2025 Sep 30;10(10):7650–60. doi: 10.1021/acssensors.5c01920 (PMC12560128; doi:10.1021/acssensors.5c01920)
Supplement: Supplementary file 1 [file se5c01920_si_001.pdf]

Supporting Information

Plasmon-Induced Graphene/Silicon Schottky Junctions for Ultrasensitive Gas Sensing

Katarzyna Drozdowska[a,*], Janusz Smulko[a], Tesfalem Welearegay[b], Lars Österlund[b], Sergey Rumyantsev[c]

[a]*Department of Metrology and Optoelectronics, Faculty of Electronics, Telecommunications, and Informatics, Gdańsk University of Technology, G. Narutowicza 11/12, 80-233, Gdańsk, Poland*

[b]*Department of Materials Science and Engineering, The Ångström Laboratory, Uppsala University, P.O. Box 35, SE-75103, Uppsala, Sweden*

[c]*Institute of High Pressure Physics PAS, 01-142 Warsaw, Poland*

*Corresponding author – katarzyna.drozdowska@pg.edu.pl

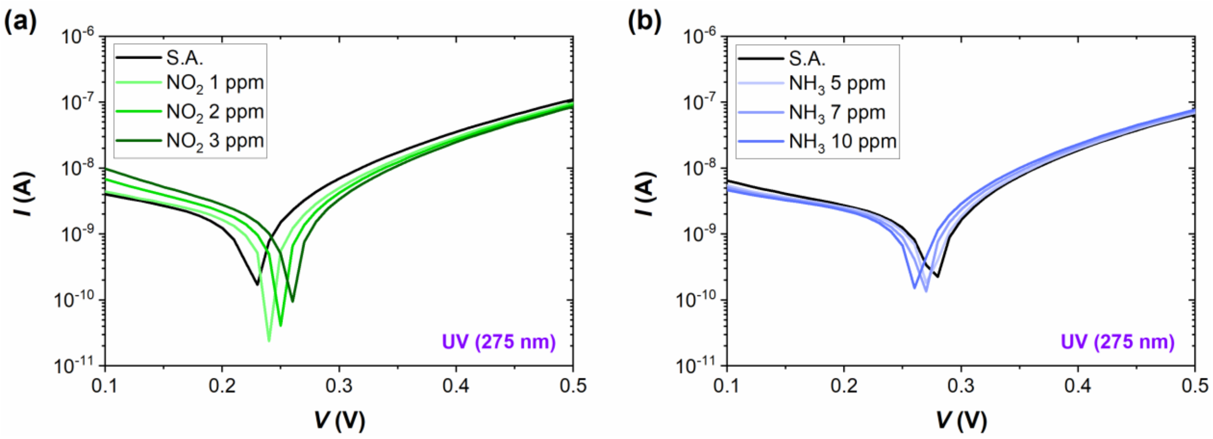

Figure S1. Current-voltage characteristics of G/Si sensor (non-decorated) response in S.A. and selected concentrations of **(a)** NO_2 (1–3 ppm) and **(b)** NH_3 (5–10 ppm) under UV irradiation of 275 nm wavelength. Further details about gas sensing by non-decorated G/Si devices can be found in [1].

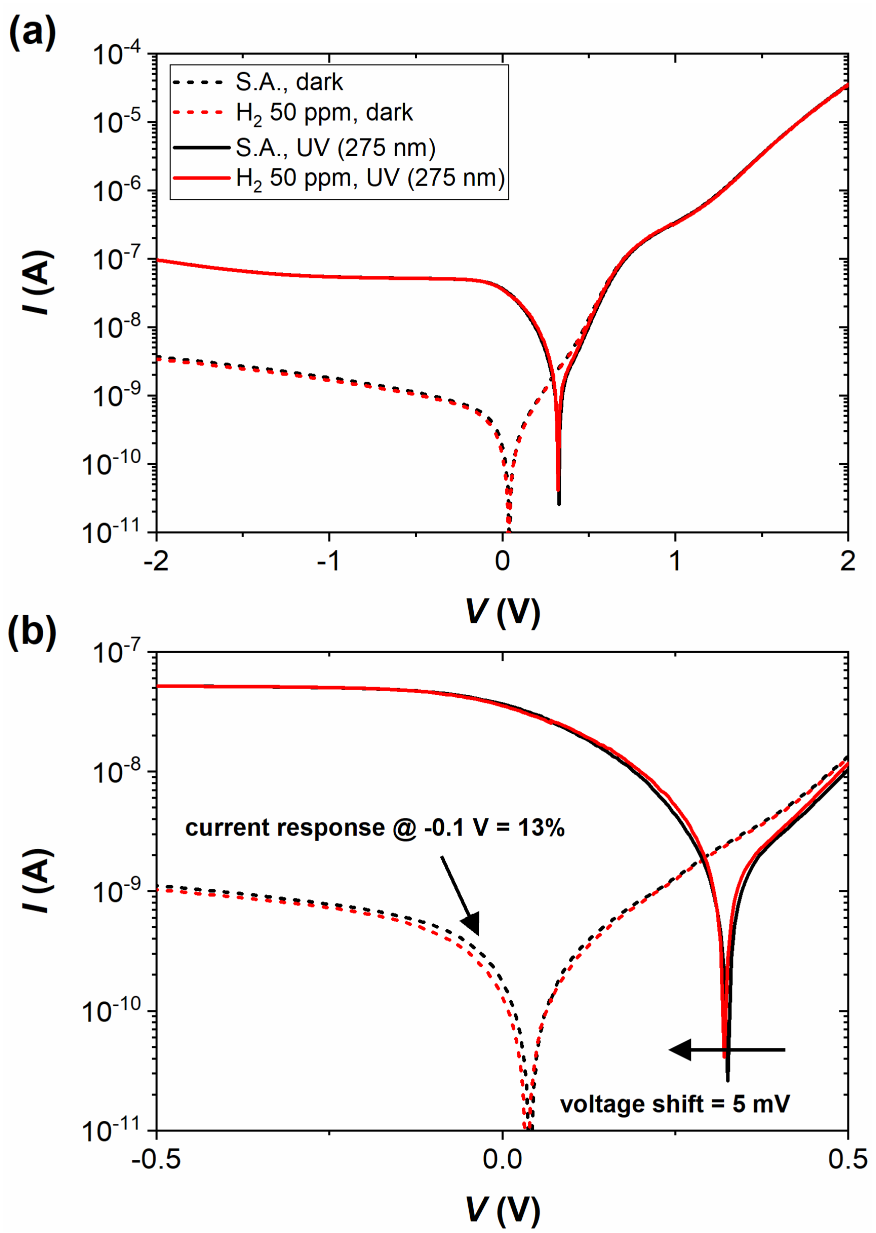

Figure S2. (a) Current-voltage (*I*-*V*) characteristics of the G/Si sensor in S.A. and 50 ppm of H_2 measured in the dark (dashed) and under UV irradiation of 275 nm wavelength (solid) and **(b)** magnified region between -0.5 V and 0.5 V showing the voltage shift under UV light (5 mV) and the current response $(I_S - I_0)/I_0$ at -0.1 V in the dark (13%).

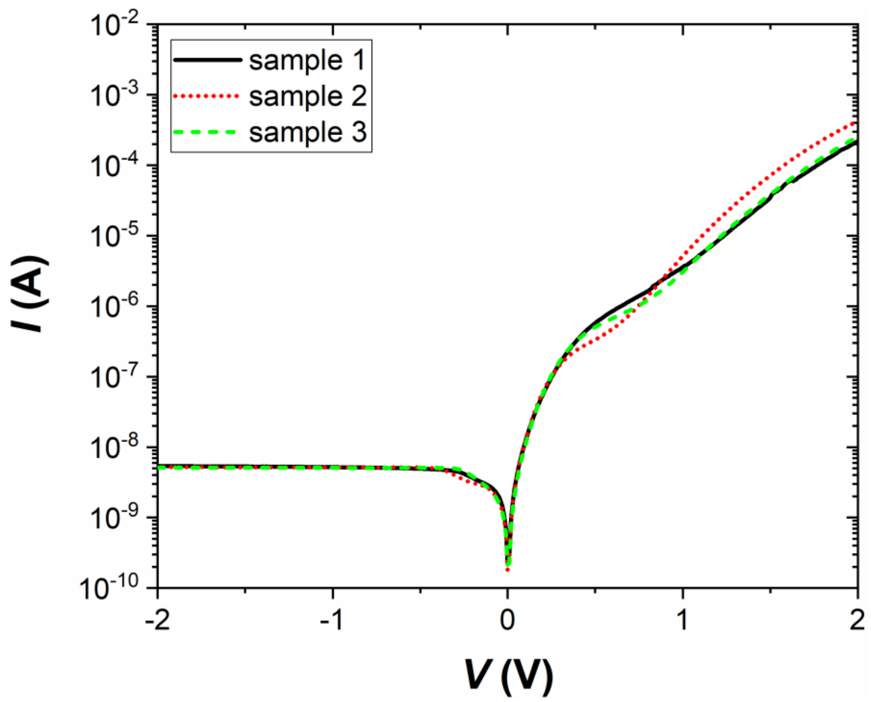

Figure S3. Current-voltage (*I*-*V*) characteristics of the G/Si sensor in laboratory air measured in the dark for three samples with Schottky diodes of the same junction dimensions (200 µm x 250 µm). Sample 1 was chosen for all gas sensing experiments presented in this work. The *I*-*V* curves show low variability in the reverse bias regime and near the zero bias current point. The differences for higher positive voltages can be ascribed to variations in the defective graphene layer, since its properties dominate this part of the *I*-*V* characteristic.

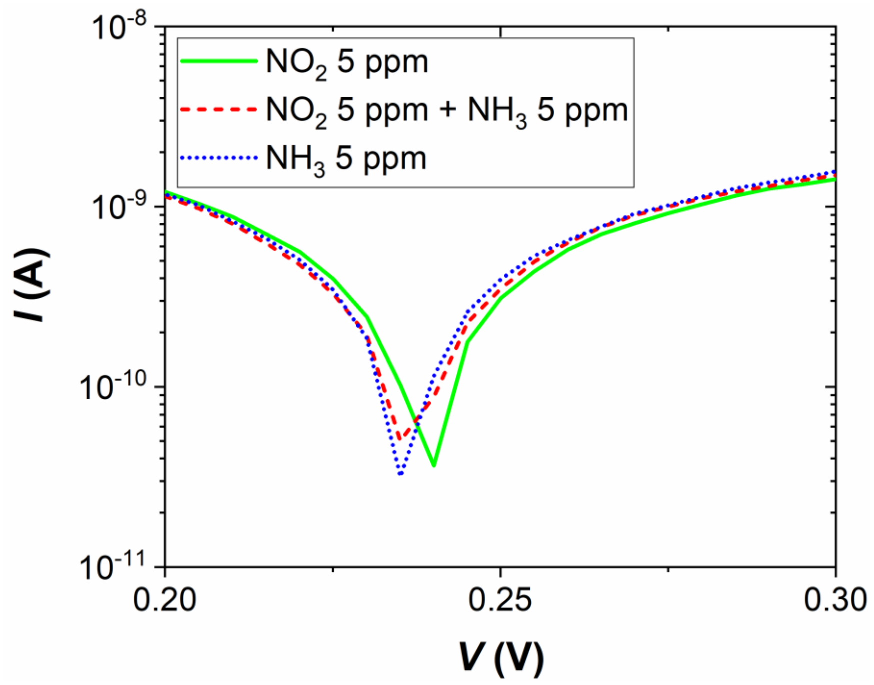

Figure S4. Close-ups of current-voltage (*I*-*V*) characteristics of the Pd-G/Si sensor measured under UV light of 275 nm wavelength for pure NO_2 (5 ppm), NH_3 (5 ppm) and the mixture of both gases. In the case of the mixed gases, the zero bias current point lies in between those obtained for pure NO_2 and NH_3, but closer to the pure NH_3 curve, showing the possibility of one gas dominating another in electrical responses.

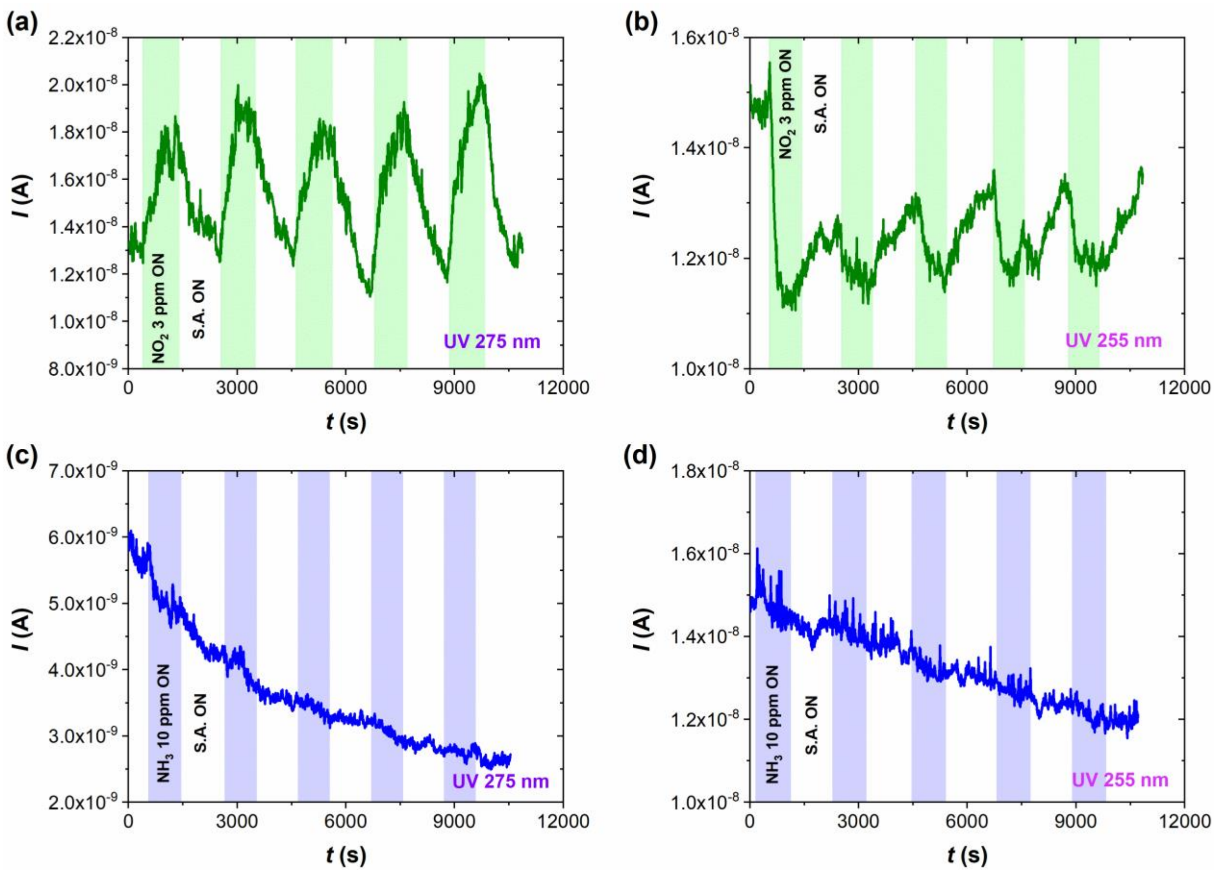

Figure S5. The sensor current responses to five consecutive cycles of 3 ppm NO_2 gas exposures under UV-light irradiation with **(a)** 275 nm, **(b)** 255 nm wavelengths, and 10 ppm NH_3 gas exposures under UV-light irradiation with **(c)** 275 nm, **(d)** 255 nm wavelengths. The voltage bias was 0.2 V and 0.1 V for measurements under UV-light irradiation with 275 nm and 255 nm wavelength, respectively. The sensors were exposed to target gases for 15 minutes, followed by 20 minutes of sensor recovery in dry S.A.

Reference

[1] K. Drozdowska, J. Smulko, A. Rehman, B. Stonio, A. Krajewska, S. Rumyantsev, and G. Cywiski, Sensing of NO_2, NH_3, and C_3H_6O by graphene-Si Schottky diode at chosen voltage biases, *Sci. Rep*. 15 (2025) 1–11, https://doi.org/10.1038/s41598-025-94473-5.
